# Supplementary material for: Extinction risk and conservation of the world’s sharks and rays
Source: eLife. 2014 Jan 21;3:e00590. doi: 10.7554/eLife.00590 (PMC3897121; doi:10.7554/eLife.00590)
Supplement: Figure 6—source data 1. — DOI: http://dx.doi.org/10.7554/eLife.00590.013 [file elife-00590-fig6-data1.docx]

Figure 6 – Source data 1

Number and IUCN Red List status of chondrichthyan species in IUCN Red List categories by family (alphabetically within each order). The number of DD species that are potentially Threatened was calculated from the proportion of data sufficient Threatened species multiplied by the number of DD species (see Section 1.7 for details). CR, Critically Endangered; EN, Endangered; VU, Vulnerable; NT, Near Threatened; LC, Least Concern; DD, Data Deficient.

| **Order** | **Family** | **CR** | **EN** | **VU** | **NT** | **LC** | **DD** | **Species** | **Threatened species (%)** | **Number of DD species that are potentially Threatened** |
| --- | --- | --- | --- | --- | --- | --- | --- | --- | --- | --- |
| Chimaeriformes | Callorhinchidae | 0 | 0 | 0 | 0 | 3 | 0 | 3 | 0 (0) | 0 |
| Chimaeriformes | Chimaeridae | 0 | 0 | 0 | 3 | 6 | 17 | 26 | 0 (0) | 0 |
| Chimaeriformes | Rhinochimaeridae | 0 | 0 | 0 | 0 | 3 | 5 | 8 | 0 (0) | 0 |
| Hexanchiformes | Chlamydoselachidae | 0 | 0 | 0 | 1 | 0 | 0 | 1 | 0 (0) | 0 |
| Hexanchiformes | Hexanchidae | 0 | 0 | 0 | 2 | 0 | 2 | 4 | 0 (0) | 0 |
| Squaliformes | Centrophoridae | 1 | 0 | 3 | 3 | 2 | 6 | 15 | 4 (26.7) | 7 |
| Squaliformes | Dalatiidae | 0 | 0 | 0 | 1 | 6 | 3 | 10 | 0 (0) | 0 |
| Squaliformes | Echinorhinidae | 0 | 0 | 0 | 1 | 0 | 1 | 2 | 0 (0) | 0 |
| Squaliformes | Etmopteridae | 0 | 0 | 0 | 0 | 21 | 21 | 42 | 0 (0) | 0 |
| Squaliformes | Oxynotidae | 0 | 0 | 1 | 0 | 0 | 4 | 5 | 1 (20) | 5 |
| Squaliformes | Somniosidae | 0 | 0 | 0 | 3 | 2 | 12 | 17 | 0 (0) | 0 |
| Squaliformes | Squalidae | 0 | 0 | 2 | 5 | 1 | 17 | 25 | 2 (8) | 6 |
| Squatiniformes | Squatinidae | 3 | 5 | 4 | 1 | 2 | 7 | 22 | 12 (54.5) | 18 |
| Pristiophoriformes | Pristiophoridae | 0 | 0 | 0 | 1 | 3 | 2 | 6 | 0 (0) | 0 |
| Rajiformes | Anacanthobatidae | 0 | 0 | 0 | 0 | 3 | 15 | 18 | 0 (0) | 0 |
| Rajiformes | Arhynchobatidae | 0 | 2 | 5 | 9 | 29 | 39 | 84 | 7 (8.3) | 13 |
| Rajiformes | Dasyatidae | 0 | 9 | 12 | 11 | 10 | 27 | 69 | 21 (30.4) | 35 |
| Rajiformes | Gymnuridae | 0 | 0 | 2 | 1 | 1 | 5 | 9 | 2 (22.2) | 5 |
| Rajiformes | Hexatrygonidae | 0 | 0 | 0 | 0 | 1 | 0 | 1 | 0 (0) | 0 |
| Rajiformes | Hypnidae | 0 | 0 | 0 | 0 | 1 | 0 | 1 | 0 (0) | 0 |
| Rajiformes | Mobulidae | 0 | 1 | 1 | 5 | 0 | 3 | 10 | 2 (20) | 3 |
| Rajiformes | Myliobatidae | 0 | 4 | 1 | 2 | 3 | 9 | 19 | 5 (26.3) | 10 |
| Rajiformes | Narcinidae | 1 | 0 | 7 | 3 | 4 | 11 | 26 | 8 (30.8) | 14 |
| Rajiformes | Narkidae | 1 | 0 | 3 | 0 | 0 | 8 | 12 | 4 (33.3) | 12 |
| Rajiformes | Platyrhinidae | 0 | 0 | 1 | 0 | 1 | 1 | 3 | 1 (33.3) | 2 |
| Rajiformes | Plesiobatidae | 0 | 0 | 0 | 0 | 1 | 0 | 1 | 0 (0) | 0 |
| Rajiformes | Potamotrygonidae | 0 | 0 | 0 | 1 | 2 | 17 | 20 | 0 (0) | 0 |
| Rajiformes | Pristidae | 7 | 0 | 0 | 0 | 0 | 0 | 7 | 7 (100) | 7 |
| Rajiformes | Rajidae | 3 | 6 | 10 | 15 | 31 | 73 | 138 | 19 (13.8) | 40 |
| Rajiformes | Rhinidae | 0 | 0 | 1 | 0 | 0 | 0 | 1 | 1 (100) | 1 |
| Rajiformes | Rhinobatidae | 1 | 3 | 11 | 7 | 6 | 17 | 45 | 15 (33.3) | 24 |
| Rajiformes | Rhinopteridae | 0 | 1 | 1 | 3 | 0 | 1 | 6 | 2 (33.3) | 2 |
| Rajiformes | Rhynchobatidae | 0 | 1 | 5 | 0 | 0 | 0 | 6 | 6 (100) | 6 |
| Rajiformes | Torpedinidae | 0 | 0 | 0 | 1 | 1 | 17 | 19 | 0 (0) | 0 |
| Rajiformes | Urolophidae | 1 | 1 | 3 | 3 | 17 | 3 | 28 | 5 (17.9) | 6 |
| Rajiformes | Urotrygonidae | 0 | 0 | 2 | 1 | 3 | 9 | 15 | 2 (13.3) | 5 |
| Rajiformes | Zanobatidae | 0 | 0 | 0 | 0 | 0 | 1 | 1 | 0 (0) | 0 |
| Heterodontiformes | Heterodontidae | 0 | 0 | 0 | 0 | 4 | 5 | 9 | 0 (0) | 0 |
| Orectolobiformes | Brachaeluridae | 0 | 0 | 1 | 0 | 1 | 0 | 2 | 1 (50) | 1 |
| Orectolobiformes | Ginglymostomatidae | 0 | 0 | 2 | 0 | 0 | 1 | 3 | 2 (66.7) | 3 |
| Orectolobiformes | Hemiscylliidae | 0 | 0 | 2 | 7 | 2 | 1 | 12 | 2 (16.7) | 2 |
| Orectolobiformes | Orectolobidae | 0 | 0 | 0 | 4 | 2 | 4 | 10 | 0 (0) | 0 |
| Orectolobiformes | Parascylliidae | 0 | 0 | 0 | 0 | 3 | 4 | 7 | 0 (0) | 0 |
| Orectolobiformes | Rhincodontidae | 0 | 0 | 1 | 0 | 0 | 0 | 1 | 1 (100) | 1 |
| Orectolobiformes | Stegostomidae | 0 | 0 | 1 | 0 | 0 | 0 | 1 | 1 (100) | 1 |
| Lamniformes | Alopiidae | 0 | 0 | 3 | 0 | 0 | 0 | 3 | 3 (100) | 3 |
| Lamniformes | Cetorhinidae | 0 | 0 | 1 | 0 | 0 | 0 | 1 | 1 (100) | 1 |
| Lamniformes | Lamnidae | 0 | 0 | 4 | 0 | 1 | 0 | 5 | 4 (80) | 4 |
| Lamniformes | Megachasmidae | 0 | 0 | 0 | 0 | 0 | 1 | 1 | 0 (0) | 0 |
| Lamniformes | Mitsukurinidae | 0 | 0 | 0 | 0 | 1 | 0 | 1 | 0 (0) | 0 |
| Lamniformes | Odontaspididae | 0 | 0 | 2 | 0 | 0 | 1 | 3 | 2 (66.7) | 3 |
| Lamniformes | Pseudocarchariidae | 0 | 0 | 0 | 1 | 0 | 0 | 1 | 0 (0) | 0 |
| Carcharhiniformes | Carcharhinidae | 5 | 3 | 6 | 21 | 9 | 7 | 51 | 14 (27.5) | 16 |
| Carcharhiniformes | Hemigaleidae | 0 | 0 | 3 | 1 | 1 | 3 | 8 | 3 (37.5) | 5 |
| Carcharhiniformes | Leptochariidae | 0 | 0 | 0 | 1 | 0 | 0 | 1 | 0 (0) | 0 |
| Carcharhiniformes | Proscylliidae | 0 | 0 | 0 | 0 | 2 | 3 | 5 | 0 (0) | 0 |
| Carcharhiniformes | Pseudotriakidae | 0 | 0 | 0 | 0 | 1 | 1 | 2 | 0 (0) | 0 |
| Carcharhiniformes | Scyliorhinidae | 1 | 2 | 5 | 8 | 34 | 88 | 138 | 8 (5.8) | 22 |
| Carcharhiniformes | Sphyrnidae | 0 | 2 | 2 | 2 | 1 | 1 | 8 | 4 (50) | 5 |
| Carcharhiniformes | Triakidae | 1 | 3 | 5 | 4 | 16 | 14 | 43 | 9 (20.9) | 13 |
|  |  |  |  |  |  |  |  |  | **Total** | **301** |
