# Supplementary material for: Extinction risk and conservation of the world’s sharks and rays
Source: eLife. 2014 Jan 21;3:e00590. doi: 10.7554/eLife.00590 (PMC3897121; doi:10.7554/eLife.00590)
Supplement: Supplementary file 1. — The Data Deficient chondrichthyan species that are potentially threatened. DOI: http://dx.doi.org/10.7554/eLife.00590.024 [file elife-00590-supp1.docx]

**Supplementary File 1 -** Potentially threatened chondrichthyan species. The probability that these Data Deficient species are threatened, *p*(THR)_DD_, was calculated using Generalized Linear Models of maximum body size and geographic distribution attributes (see Table 3 in the main text for key to models). The table is ordered by the number times the species was classified as threatened *p*(THR)_DD_ ≥ 0.5 and weighted mean threat score. The average *p*(THR)_DD_ was calculated based on the relative importance of variables (Figure 3).

| **Species name** | **Model 1 *p*(THR)_DD_** | **Model 2 *p*(THR)_DD_** | **Model 3 *p*(THR)_DD_** | **Model 4 *p*(THR)_DD_** | **Weighted mean *p*(THR)_DD_** | **TimesTHR** |
| --- | --- | --- | --- | --- | --- | --- |
| *Somniosus pacificus* | 0.816 | 0.835 | 0.735 | 0.787 | 0.736 | 4 |
| *Ginglymostoma cirratum* | 0.735 | 0.785 | 0.826 | 0.830 | 0.735 | 4 |
| *Aetobatus guttatus* | 0.722 | 0.777 | 0.792 | 0.833 | 0.722 | 4 |
| *Pastinachus sephen* | 0.680 | 0.752 | 0.829 | 0.798 | 0.708 | 4 |
| *Dasyatis rudis* | 0.677 | 0.538 | 0.920 | 0.941 | 0.705 | 4 |
| *Megachasma pelagios* | 0.778 | 0.733 | 0.806 | 0.705 | 0.703 | 4 |
| *Dasyatis thetidis* | 0.722 | 0.777 | 0.742 | 0.798 | 0.703 | 4 |
| *Carcharhinus amboinensis* | 0.649 | 0.733 | 0.809 | 0.770 | 0.686 | 4 |
| *Notorynchus cepedianus* | 0.656 | 0.738 | 0.766 | 0.752 | 0.675 | 4 |
| *Dasyatis pastinaca* | 0.624 | 0.718 | 0.714 | 0.719 | 0.643 | 4 |
| *Glaucostegus halavi* | 0.536 | 0.664 | 0.759 | 0.787 | 0.633 | 4 |
| *Rhizoprionodon longurio* | 0.511 | 0.648 | 0.767 | 0.759 | 0.620 | 4 |
| *Carcharhinus porosus* | 0.505 | 0.644 | 0.744 | 0.702 | 0.600 | 4 |
| *Orectolobus hutchinsi* | 0.503 | 0.643 | 0.663 | 0.730 | 0.585 | 4 |
| *Carcharhinus cautus* | 0.505 | 0.644 | 0.612 | 0.609 | 0.550 | 4 |
| *Sphyrna media* | 0.505 | 0.644 | 0.612 | 0.597 | 0.547 | 4 |
| *Myliobatis tobijei* | 0.505 | 0.644 | 0.604 | 0.603 | 0.546 | 4 |
| *Taeniurops grabata* | 0.624 | 0.573 | 0.596 | 0.541 | 0.543 | 4 |
| *Carcharhinus altimus* | 0.664 | 0.527 | 0.562 | 0.546 | 0.534 | 4 |
| *Torpedo nobiliana* | 0.548 | 0.672 | 0.531 | 0.517 | 0.529 | 4 |
| *Bathyraja aguja* | 0.755 | 0.366 | 0.901 | 0.940 | 0.676 | 3 |
| *Somniosus antarcticus* | 0.792 | 0.462 | 0.557 | 0.636 | 0.565 | 3 |
| *Paragaleus leucolomatus* | 0.400 | 0.574 | 0.706 | 0.777 | 0.563 | 3 |
| *Paragaleus pectoralis* | 0.485 | 0.631 | 0.652 | 0.647 | 0.559 | 3 |
| *Rhinobatos leucospilus* | 0.452 | 0.610 | 0.623 | 0.674 | 0.544 | 3 |
| *Heterodontus francisci* | 0.456 | 0.612 | 0.591 | 0.630 | 0.529 | 3 |
| *Torpedo sinuspersici* | 0.471 | 0.622 | 0.581 | 0.565 | 0.520 | 3 |
| *Torpedo panthera* | 0.431 | 0.596 | 0.596 | 0.589 | 0.512 | 3 |
| *Narcine entemedor* | 0.425 | 0.591 | 0.598 | 0.594 | 0.511 | 3 |
| *Torpedo fairchildi* | 0.573 | 0.583 | 0.455 | 0.544 | 0.499 | 3 |
| *Mustelus norrisi* | 0.405 | 0.577 | 0.594 | 0.562 | 0.495 | 3 |
| *Dasyatis navarrae* | 0.395 | 0.570 | 0.570 | 0.605 | 0.494 | 3 |
| *Orectolobus japonicus* | 0.425 | 0.591 | 0.538 | 0.506 | 0.479 | 3 |
| *Zapteryx exasperata* | 0.402 | 0.575 | 0.516 | 0.565 | 0.476 | 3 |
| *Sympterygia bonapartii* | 0.380 | 0.559 | 0.556 | 0.552 | 0.474 | 3 |
| *Rhinobatos glaucostigma* | 0.383 | 0.561 | 0.548 | 0.544 | 0.471 | 3 |
| *Gogolia filewoodi* | 0.342 | 0.531 | 0.545 | 0.589 | 0.462 | 3 |
| *Nasolamia velox* | 0.505 | 0.460 | 0.509 | 0.514 | 0.460 | 3 |
| *Heterodontus mexicanus* | 0.330 | 0.522 | 0.566 | 0.529 | 0.451 | 3 |
| *Squatina dumeril* | 0.508 | 0.646 | 0.451 | 0.478 | 0.486 | 2 |
| *Squatina africana* | 0.456 | 0.612 | 0.486 | 0.529 | 0.483 | 2 |
| *Mustelus manazo* | 0.446 | 0.606 | 0.506 | 0.485 | 0.475 | 2 |
| *Mustelus palumbes* | 0.452 | 0.610 | 0.464 | 0.518 | 0.474 | 2 |
| *Rhinobatos prahli* | 0.362 | 0.269 | 0.744 | 0.694 | 0.472 | 2 |
| *Bathyraja ishiharai* | 0.458 | 0.155 | 0.700 | 0.737 | 0.464 | 2 |
| *Raja maderensis* | 0.359 | 0.544 | 0.498 | 0.606 | 0.462 | 2 |
| *Mustelus griseus* | 0.411 | 0.470 | 0.585 | 0.525 | 0.461 | 2 |
| *Mustelus punctulatus* | 0.397 | 0.572 | 0.511 | 0.478 | 0.456 | 2 |
| *Heterodontus omanensis* | 0.302 | 0.232 | 0.691 | 0.755 | 0.448 | 2 |
| *Dasyatis sinensis* | 0.364 | 0.548 | 0.478 | 0.534 | 0.445 | 2 |
| *Squalus raoulensis* | 0.339 | 0.183 | 0.671 | 0.748 | 0.438 | 2 |
| *Zanobatus schoenleinii* | 0.409 | 0.421 | 0.546 | 0.514 | 0.437 | 2 |
| *Hemitriakis complicofasciata* | 0.400 | 0.277 | 0.579 | 0.555 | 0.415 | 2 |
| *Heterodontus quoyi* | 0.302 | 0.423 | 0.540 | 0.531 | 0.413 | 2 |
| *Rhinobatos punctifer* | 0.380 | 0.401 | 0.502 | 0.503 | 0.412 | 2 |
| *Centrophorus seychellorum* | 0.358 | 0.148 | 0.645 | 0.665 | 0.411 | 2 |
| *Squalus lalannei* | 0.356 | 0.147 | 0.643 | 0.667 | 0.410 | 2 |
| *Chiloscyllium burmensis* | 0.288 | 0.271 | 0.581 | 0.568 | 0.390 | 2 |
| *Rajella sadowskii* | 0.345 | 0.136 | 0.625 | 0.570 | 0.381 | 2 |
| *Apristurus fedorovi* | 0.324 | 0.129 | 0.604 | 0.628 | 0.381 | 2 |
| *Asymbolus funebris* | 0.241 | 0.155 | 0.585 | 0.559 | 0.349 | 2 |
| *Psammobatis parvacauda* | 0.202 | 0.152 | 0.553 | 0.615 | 0.342 | 2 |
| *Cephaloscyllium zebrum* | 0.244 | 0.130 | 0.556 | 0.584 | 0.342 | 2 |
| *Cirrhoscyllium formosanum* | 0.220 | 0.166 | 0.582 | 0.518 | 0.338 | 2 |
| *Mustelus mosis* | 0.505 | 0.440 | 0.473 | 0.454 | 0.434 | 1 |
| *Echinorhinus brucus* | 0.671 | 0.356 | 0.440 | 0.398 | 0.434 | 1 |
| *Mustelus sinusmexicanus* | 0.488 | 0.389 | 0.448 | 0.521 | 0.425 | 1 |
| *Mustelus widodoi* | 0.431 | 0.320 | 0.475 | 0.600 | 0.416 | 1 |
